# The active commuting route environment scale (ACRES): development and evaluation

**DOI:** 10.1186/1479-5868-7-58

**Published:** 2010-07-07

**Authors:** Lina Wahlgren, Erik Stigell, Peter Schantz

**Affiliations:** 1 The Research Unit for Movement, Health and Environment, The Åstrand Laboratory, GIH - The Swedish School of Sport and Health Sciences, SE-114 86 Stockholm, Sweden; 2 School of Health and Medical Sciences, Örebro University, SE-701 82 Örebro, Sweden; 3 Department of Health Sciences, Mid Sweden University, SE-831 25 Östersund, Sweden

## Abstract

**Background:**

Route environments can be a potentially important factor in influencing people's behaviours in relation to active commuting. To better understand these possible relationships, assessments of route environments are needed. We therefore developed a scale; the Active Commuting Route Environment Scale (ACRES), for the assessment of bicyclists' and pedestrians' perceptions of their commuting route environments. Here we will report on the development and the results of validity and reliability assessments thereof.

**Methods:**

Active commuters (n = 54) were recruited when they bicycled in Stockholm, Sweden. Traffic planning and environmental experts from the Municipality of Stockholm were assembled to form an expert panel (n = 24). The active commuters responded to the scale on two occasions, and the expert panel responded to it once. To test criterion-related validity, differences in ratings of the inner urban and suburban environments of Greater Stockholm were compared between the experts and the commuters. Furthermore, four items were compared with existing objective measures. Test-retest reproducibility was assessed with three types of analysis: order effect, typical error and intraclass correlation.

**Results:**

There was a concordance in sizes and directions of differences in ratings of inner urban and suburban environments between the experts and the commuters. Furthermore, both groups' ratings were in line with existing objectively measured differences between the two environmental settings. Order effects between test and retest were observed in 6 of 36 items. The typical errors ranged from 0.93 to 2.54, and the intraclass correlation coefficients ranged from 'moderate' (0.42) to 'almost perfect' (0.87).

**Conclusions:**

The ACRES was characterized by considerable criterion-related validity and reasonable test-retest reproducibility.

## Background

Active transport is a behaviour that could favour increasing the level of physical activity within the population. In the interest of understanding active physical behaviours, the ecological model has emphasized the environment as a potentially important factor. Furthermore, it emphasizes that both people's perceptions and more objectively assessed aspects of the environments are likely to influence people's behaviours [1]. In line with this view, mixed land use and residential density, street connectivity and physical infrastructure, such as pavements, are factors that have been found to be related to levels of physical activity in general in population samples [cf. [2]].

Physical activity includes different domains, such as exercise, recreational activities, household and occupational activities and active transport [1]. Pioneers in the research field of physical activity and the environment have pointed out the need for distinguishing particular types and purposes of physical activity and their conceivable relation to the specific environments in which they occur [e.g. [3,4]]. Despite these early suggestions little has been done. Active commuting by either bicycle or foot is such a particular physical activity, and the associated route setting is such a specific environment. It is therefore of interest to study whether the route environments *per se *may affect different levels of perception and behaviour related to active commuting.

Active commuting is normally a repetitive behaviour along a specific route. This makes the active commuters very familiar with their individual route environments. Their perceptions of the route environments can therefore be considered to be relevant, and possibly further our understanding of the influence route environments may have on active transport in general. Given this background, it is essential to be able to assess different components of active commuting route environments. The environment can be assessed more or less objectively with, e.g. the Geographical Information System (GIS) or audit tools [5,6], or subjectively with e.g. self-reports. Some questionnaires have been developed to subjectively assess the neighbourhood environment possibly associated with physical activity [7-11]. Psychometric properties have been documented for some of these questionnaires. The reported reliability is generally reasonable [3,7,9,10,12-16]. Validity has also been reported, but less frequently [3,10,17-19].

As mentioned, these questionnaires deal primarily with the neighbourhood, often defined as the area within a 10 to 15-minute walk from your home [e.g. [7]], or similar specifications. This local area might not, however, capture important environmental facets connected to physical activity that takes place elsewhere. Active commuting, for instance, often involves an extended environment compared to the neighbourhood [20,21]. We have therefore developed a scale, named the Active Commuting Route Environment Scale (ACRES), for the assessment of bicyclists' and pedestrians' perceptions of different variables in their commuting route environment. Interestingly, at about the same period of time, Titze et al. [22] also developed a self-report tool that considers bicycling and route environments. The two instruments were developed independently of each other, without either one of the involved persons knowing about the other process. Apart from differences in items related to route environments, Titze et al. [22] make use of Likert scales, which lead to other statistical analyses than those that the ACRES enables.

The ACRES can be used for different purposes. Our primary aim, however, in the development of this instrument, which has 15-point response scales, was to enable evaluations of relations between possible predictor variables, such as perceptions of congestion, greenery, exhaust fumes and noise, and the following outcome variables: (a) perception of traffic safety; (b) perception of whether the overall route environment stimulates or hinders active commuting; and (c) levels of active commuting (distance or time, and trip frequency). Since perceptions of traffic unsafety have been reported to be a major hindrance to active transport by bicycling [cf. [23]], it is important to understand which components might explain that perception. Other components in the route environments might be related to stimulating the active commuting in and of itself. Those components might or might not, however, be related to levels of active commuting. Our working hypothesis is that these three different outcomes are dependent on at least partially different predictor variables.

Here we will describe the development of the ACRES, and report on its validity and reliability. Validity was assessed as criterion-related validity and based on differences between inner urban and suburban environments, in existing objective measures and in ratings of an expert panel as well as of active commuters. Reliability was assessed as test-retest reproducibility among active commuters.

## Methods

### Recruitment of commuting participants

Sampling of commuting participants was aimed at reasonable representativity for active commuters in the region during the sampling period. Active commuters are a small group within the general population and, furthermore, for the validity assessment approach in our study we needed participants who commuted in both the inner urban and suburban parts of Greater Stockholm (see below). Therefore, it was not possible, in practical terms, to recruit the participants from a random population sample. Instead, the participants were recruited between 7 and 9 a.m. in mid-November, 2005, while they were walking or bicycling into or in the inner urban area of Stockholm, Sweden. The recruitment took place as they either slowed down at one of four bridges or stopped at a traffic light on one arterial road. For geographical reasons, three of these places of recruitment (two bridges and one arterial road) were focal centres for active commuters entering the inner urban part of Stockholm from three different parts of the surrounding suburban landscape. People living in these three different suburban areas represent slightly different sociodemographic characteristics.

An invitation to participate together with a reply coupon was handed to 589 persons. Overall, 214 coupons were returned in due time. The participants were then divided into two subgroups, one of which was used in this study (n = 100). The other group was used for a reproducibility study of another questionnaire. Bicyclists and dual mode performers, who sometimes walked and sometimes rode a bicycle, were selected for the study (n = 83). Only data on bicycle commuting have been used.

Eligibility criteria included: (a) being at least 20 years old; (b) living in the Stockholm County, excluding the municipality of Norrtälje; and (c) walking and/or bicycling the whole way to one's place of work or study at least once a year. In the invitation to participate, it was emphasized that people with short commuting distances were also welcome to participate. The reason for including people with less frequent active commuting behaviours, as well as with short route distances, was to include a wide range of commuting behaviours.

The Ethics Committee of the Karolinska Institute approved the study. The participants gave their informed consent. They were not paid for their participation, but they received a lottery ticket and a bicycling map as a token of gratitude and as an incentive, together with the dispatch of a second letter.

### Commuting participants and procedure

A questionnaire and a letter with information was sent home to each participant during November to December, 2005 (n = 83). Participants were asked to return the complete questionnaire by mail using a prepaid return envelop, and 73 did so. About two weeks after the questionnaire had been returned, the participants received a second questionnaire identical to the first one. Fifty-six participants returned the retest questionnaire. After cleansing and editing the data, a total of 54 participants (women, n = 20) were included in the analyses. Of these, 49 were bicyclists and 5 were dual mode performers. Based on self-reported data, the mean number of their active commuting trips per year was 339 ± 89 (± SD, n = 35). For November and December, the mean numbers of active commuting trips per week were approximately 8 and 5 (n = 42 and 41), respectively. The 54 participants yielded data in the following subgroups: (a) bicycling in an inner urban environment (n = 53) and (b) bicycling in a suburban environment (n = 45). Of these participants, 44 (women, n = 16) yielded data in both inner urban and suburban environments. For further descriptive characteristics of the participants, see Table 1.

**Table 1:** Descriptive characteristics of the commuting participants and experts

	Commuters		Experts (n = 23-24)
		
Characteristic	Women (n = 19-20)	Men (n = 34)	
Age in years, mean ± SD	40.8 ± 8.9	47.3 ± 10.3	43.8 ± 9.2
Weight in kg, mean ± SD	61.1 ± 6.1	77.1 ± 7.6	-
Height in cm, mean ± SD	169.4 ± 4.4	180.0 ± 6.2	-
Body mass index, mean ± SD	21.3 ± 2.1	23.8 ± 2.2	-
Gainful employment, %	100*	97	-
Having a driver's license, %	95	94	96†
Usually access to a car, %	70	82	83†
Educated at university level, %	75	74	100
An income above 25.000 SEK‡ a month, %	50	82	100†
Overall physical health as either good or very good, %	100	88	-
Overall mental health as either good or very good, %	90	91	-

Values are based on self-reports.

*n = 19, i.e. one missing value.

†n = 23, i.e. one missing value.

‡SEK = Swedish crown: Year 2005 and 2009: €1 ≈ 10 SEK; US$1 ≈ 8 and 7 SEK, respectively.

In order not to influence the results, the participants were not informed about the purpose of the study until the second dispatch. They were then informed that the duplicating procedure was undertaken to enable evaluation of the certainty of the study's results.

In some cases, snow was falling between the two test occasions. In such cases, the participants were instructed to recall the conditions of the first test occasion regarding the items that could have changed due to the snow, and to report them also on the retest occasion.

### Questionnaire

The ACRES is a module in the second of two questionnaires named the Physically Active Commuting in Greater Stockholm Questionnaires (PACS Q). Both PACS Q1 and Q2 are self-administered questionnaires in Swedish, based on self-reports and developed by the last two authors. The questionnaires were pre-tested on a small convenience sample of academic staff members.

**Figure 1:**
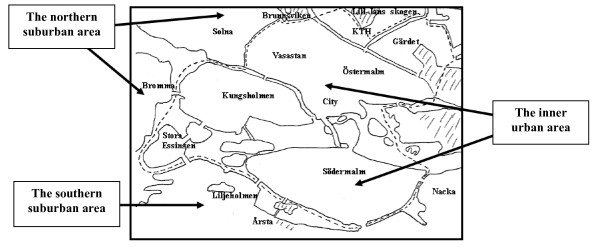
**The drawn map that was included in the ACRES instructions to the participants**. The dashed line distinguishes the inner urban and the suburban areas of Greater Stockholm. The lake Mälaren and inner parts of the Baltic Sea in the Stockholm archipelago create a natural separation between the southern and northern suburban and rural areas.

The PACS Q2 contains about 70 items, whereof the ACRES consists of 18 items for the assessment of bicyclists' perceptions of their self-chosen commuting route potentially associated with active commuting (see Table 2), and 15, fundamentally identical, items for the assessment of the pedestrians' perceptions. Each item considers the inner urban area of Stockholm, the capital of Sweden, and the suburban as well as rural areas surrounding it, within Stockholm County, separately. The questionnaire instructions include a drawn map that distinguishes the inner urban area from the surrounding area (Figure 1). The participants are asked to differentiate between their experiences when their active commuting route is in the inner urban area and when it is in the surrounding suburban as well as rural area. All items have two identical parallel response lines. One line refers to the inner urban area and the other to the suburban as well as rural area. If the participants cycle or walk in both environments, they are asked to mark both lines. If the participants, for instance, first cycle in the southern suburban area, then cross into the inner urban area and finish their route in the northern suburban area, they are asked to give an average rating for both suburban areas of the route.

**Table 2:** The Active Commuting Route Environment Scale (ACRES) for bicyclists

	15-point response scale	
	
Question	1	15
1. How do you experience the environment on the whole along the route?	Very bad	Very good
2. Do you think that, on the whole, the environment you cycle in stimulates/hinders your commuting?	Hinders a lot	Stimulates a lot
3. How do you find the exhaust fume levels along your route?	Very low	Very high
4. How do you find the noise levels along your route?	Very low	Very high
5. How do you find the flow of motor vehicles (number of cars) along your route?	Very low	Very high
6. How do you find the speeds of motor vehicles (taxis, lorries, ordinary cars, buses) along your route?	Very low	Very high
7. How do you find other cyclists' speeds along your route?	Very low	Very high
8. How do you as a cyclist find the congestion levels in mixed traffic, caused by all types of vehicles, along your route?	Very low	Very high
9. How do you find the congestion levels caused by the number of cyclists on the cycle paths/cycle lanes along your route?	Very low	Very high
10. How do you find the occurrence of conflicts between you as a cyclist and other road users (including pedestrians) along your route?	Very low	Very high
11. About how large a part of your route consists of cycle paths/cycle lanes/roads separated from motor-car traffic?	0%	100%*
12. How unsafe/safe do you feel in traffic as a cyclist along your route?	Very unsafe	Very safe
13. How do you find the availability of greenery (natural areas, parks, planted items, trees) along your route?	Very low	Very high
14. How ugly/beautiful do you find the surroundings along your route?	Very ugly	Very beautiful
15. To what extent do you feel that your cycle trip is made more difficult by the course of the route? For example a course with many sharp turns, detours, changes in direction, side changeovers etc.	Very little	Very much
16. To what extent do you feel that your cycle trip is made more difficult by hilliness? Base this on the route to and from your place of work/study.	Very little	Very much
17. To what extent do you feel that your progress in traffic is worsened by the number of red lights during your trip to your place of work/study?	Very little	Very much
18. How short/long do you experience your route to be?	Very short	Very long

Note that this is a translation of the original ACRES in Swedish.

*11-point scale.

To simplify understanding of the items, we have divided them into: (a) the physical environment; (b) the traffic environment; and (c) the social environment. The following items are included in the physical environment: *bicycle paths *(#11) (not for pedestrians), *greenery *(#13), *ugly or beautiful *(#14), *course of the route *(#15), *hilliness *(#16), *red lights *(#17) and *short or long *(#18). They represent non-moving aspects. The following items are included in the traffic environment: *exhaust fumes *(#3), *noise *(#4), *flow of motor vehicles *(#5), *speeds of motor vehicles *(#6), *speeds of bicyclists *(#7) (not for pedestrians), *congestion: all types of vehicles *(#8) (not for pedestrians) and *congestion: bicyclists/pedestrians *(#9). They represent moving aspects. The following item is included in the social environment: *conflicts *(#10). It represents relationships between road users. All items are meant to operate independently. The remaining three items, namely, *on the whole *(#1), *hinders or stimulates *(#2) and *traffic: unsafe or safe *(#12), are regarded as outcome variables. All the other items are regarded as predictor variables believed to be potentially important for the outcome variables. The numbers specified in parentheses indicate the order in the questionnaire; see Table 2.

Fifteen-point response scales, with adjectival opposites, ranging from 1 to 15, corresponding to e.g. 'very low' and 'very high', are used, with the exception of one item. The item *bicycle paths *has an 11-point response scale ranging from 0% (0) to 100% (10). The 15-point response scales feature a numbered continuous line, i.e. whole numbers from 1 to 15, with number 8 as a neutral option in the middle, labelled, e.g., 'neither low nor high'.

In the questionnaire instructions, the participants are asked to recall and rate their overall experience of their self-chosen route environments based on their active commuting to their place of work or study during the previous two weeks. At no point were the participants informed about the intent of the ACRES.

### Development of the environmental scale - issues related to construct and content validity

The development of the environmental scale, ACRES, was undertaken by the last two authors, and was basically carried out in line with the procedure for the development of the Neighborhood Environment Walkability Scale (NEWS) [10]. It was influenced by published research literature in the field, as well as by the last two authors' many years of bicycling commuting experiences and, furthermore, by one of the authors' professional experiences from working with bicycling advocacy and promotion issues in the region of Stockholm. The outcome variable pertaining to whether the environment on the whole is perceived as stimulating or hindering physically active commuting (*hinders or stimulates*) was formulated to be specific for the particular physical activity behaviour studied [4]. It was complemented with a more generally formulated outcome variable concerning how the environment on the whole along the commuting route is perceived (*on the whole*). The outcome variable *traffic: unsafe or safe *was prompted by the fact that feelings of unsafeness have been reported as an important hindrance to cycling [cf. [23]].

The predictor variables *flow of motor vehicles *and *speeds of motor vehicles *were chosen based on a mixture of inputs, including a conceptual framework developed by Pikora et al. [24]. The included composite expressions of these two items were *noise*, *exhaust fumes *and *congestion: all types of vehicles*. The latter item may also be influenced by the item *congestion: bicyclists*, although it is related to bicyclists in bicycle paths or lanes. However, *congestion: bicyclists *can also be regarded as an indicator of the flow of bicyclists in general. The item *congestion: bicyclists *was also prompted by concerns expressed by civil servants dealing with bicycle traffic at the traffic unit of the Municipality of Stockholm (personal communication from Krister Isaksson) in relation to an increasing flow of bicyclists. Frequently noted complaints regarding bicyclist behaviours by citizens, addressed as letters from 'Readers' or 'Opinions' in the two major Stockholm morning newspapers were among the reasons for the items *speeds of bicyclists *and *conflicts*. The item *bicycle paths *was chosen because it is an often suggested infrastructure investment in policy documents aimed at increasing bicycling. Furthermore, in a population study in the Municipality of Stockholm, it has been cited as an issue influencing the willingness to cycle more [25]. The inclusion of the item *greenery *was prompted by the fact that natural elements appear to be a modifier of stress and mood states [cf. [26,27]]. Greenery can be anticipated to be a component of the item regarding aesthetics (*ugly or beautiful*), which, however, can be a composite expression of other sources of beauty as well. *Ugly or beautiful *merited inclusion also based on findings regarding the local neighbourhood and levels of walking [28]. The items *course of the route*, *hilliness *and *red lights *were related to the theories of space syntax [29,30]. The item *short or long *was seen as a potentially important perception in relation to the outcome variable *hinders or stimulates*. Note that all items in ACRES can vary independently of each other, and that the scale was developed to enable evaluations of potential separate effects of individual items and the relations between them.

To the best of our knowledge, this is the first time that items related to space syntax have been integrated in this type of scale. We will therefore give a background on the items connected with space syntax. The theory behind it all states that the configuration of the street network in and of itself is a strong movement generator in relation to walking. It postulates that the fewer the number of direction changes that the street network requires a person to make to reach a certain destination, the more the street configuration will stimulate movement [30]. Particularly when human movements take place in street networks, the route taken can easily be described in terms of so-called axial lines. Each axial line represents the horizontal straight line that a moving object can take before it has to make an angular turn to be able to progress. The shift in direction can lead into, e.g., another street or be necessary due to the fact that the street is not straight. The item *course of route *in the ACRES relates to this issue.

The axial lines are horizontal representations, but they also stand for distinct visual axial lines and spaces. These change with the direction of the movement. Even vertical movement may contribute to changes in visual axial lines. For example, a larger hill along a straight road breaks the visual continuity enabled by the characteristics of a straight road. When the top of the hill is approached a new visual axial line is disclosed. For this reason, hilliness is an item of its own. Thus, it is possible to distinguish two different causes for the number of visual axial lines along a route. This enables one to distinguish a potential separate effect of hilliness on the number of axial visual lines from that of the horizontal axial lines *per se*. Another important reason for the inclusion of the item *hilliness *is its stated impact of hindering movements due to greater demands on effort [e.g. [23]]. Finally, the number of red lights along a route may possibly have an independent effect on hindering or stimulating movement, as well as on the perception of traffic safety, and is therefore an item of itself. Thus, the three items *course of the route*, *hilliness *and *red lights *will jointly facilitate an evaluation of the theories of space syntax concerning movement generation, within the whole concept that the ACRES represents.

As indicated above, one aim of the development of the ACRES was to enable the evaluation of relations between and within the predictor and outcome variables. This affected the choice of items, including the response scales. An additional input was the changes in motorized traffic flows expected to occur with the introduction of a congestion tax at the limits of the Stockholm inner urban area in 2006 [31]. This could potentially lead to changes in different environmental variables connected with the traffic environment, as well as with a changeover to more active transport. These changes were considered to be interesting to examine in terms of perceptions by active commuters. Some of the anticipated changes, e.g. in exhaust fume levels, were in the order of 10% [32]. This was the reason for choosing response scales which, in principle, have the potential to capture changes of finer distinction. If the anchors of the response scale, 1 to 15, were viewed as 0 and 100%, respectively, each of the 14 scale steps could be considered to represent about 7.1% and thus, in principle be useful for assessing responses to perceived changes of rather small sizes.

### Validation of the environmental scale

Validity assessments can be complicated when no objective data exist or are difficult to gather for comparison. This is indeed the case for validation of peoples' perceptions of active commuting route environments. Furthermore, perception is an individually dependent and, in many cases, relative issue. Since the ACRES addresses the inner urban and the suburban environments separately (see above), we considered that one possible approach was to use some expected differences between the two environments for criterion-related validation. Therefore, the 'known group difference method' [33] served as a model. In our case, known and existing objectively measured differences between inner urban and suburban environments of Greater Stockholm, corresponding to the four items, *exhaust fumes*, *noise*, *congestion: all types of vehicles *and *greenery*, were used for comparisons of direction of differences (see below). The commuting participants who provided data in both inner and suburban environments were used for the criterion-related validity assessments. Furthermore, an expert panel was assembled, selected on the basis of a solid knowledge of both inner urban and suburban traffic environments of Greater Stockholm, and was therefore considered to be appropriate for the validation of the ACRES. The panel members received a modified version of the ACRES (see below). First, the directions of differences of ratings of the experts and the commuters for the two different environments were compared with the directions of differences of existing objective measures of these environments. Thereafter, directions and sizes of potential differences in the ratings of inner urban and suburban environments were compared between the experts and the commuters.

#### Existing objective measures

Ratings of the four items *exhaust fumes*, *noise*, *congestion: all types of vehicles *and *greenery *were compared with directions of differences in existing objective measures of the inner urban and suburban areas of Greater Stockholm. An objective indication of the difference in congestion levels between these settings is the introduction of the road traffic congestion charges for the inner urban area of Stockholm in January, 2006 [31,32]. However, the traffic environment in this part of Stockholm is still more intense than in the suburban area [32,34]. Differences in levels of noise and exhaust fumes between the environments are shown by a higher density of streets having high levels of noise [35], as well as higher levels of particular matters (e.g. PM10) and nitrogen oxides [36] in the inner urban area. Finally, most streets in the inner urban area of Stockholm are lacking green elements such as trees, and other forms of greenery are sparse. On the other hand, green elements are quite frequent in the suburban areas. This difference is evident in a visual inspection using an aerial view over these two environments, and it is also apparent in biotope mappings of Stockholm [37].

#### The expert panel

An expert panel was assembled to assess the inner urban and the suburban environments of Greater Stockholm. The Municipality of Stockholm includes both of these types of environment. Therefore, based on the recommendation of leading civil servants working with traffic planning for bicycling and environmental issues, 32 relevant employees of the Municipality of Stockholm were chosen to be part of the expert panel. These 32 experts were employed at the exploitation, traffic, city planning, and environment units, respectively, of the Municipality of Stockholm. A letter introducing the study and inviting the experts to participate was sent, together with a questionnaire, to the experts in September, 2009. The experts gave their informed consent. They received cinema tickets as an incentive after returning the questionnaire. The items in their questionnaire were modified versions of the items in the ACRES assessing bicyclists' perceptions. One item, *short or long*, was not included in the expert questionnaire. The experts were asked to assess: (a) the overall route environments for bicyclists commuting in Greater Stockholm and commuting bicyclists as a whole group and (b) inner urban and suburban areas separately. They were also asked to comment on the items in the ACRES and encouraged to name factors of importance in the environment of bicycling commuters that they felt were missing (see Discussion). Twenty-eight experts returned the questionnaire, and data from a total of 24 experts (women, n = 11) could be included in the analyses (1 did not complete the questionnaire and 3 misinterpreted the instructions). Based on self-reported data, 10 of the participants usually commuted to work by bicycle all the year round, and 4 did so during the summer half-year. For further descriptive characteristics of the experts, see Table 1.

### Statistical Analyses

#### Statistics

Statistical analyses of differences between men and women, test and retest, and inner urban and suburban environments, respectively, were initially performed using both parametric (Student's independent or paired t-test) and non-parametric tests (Mann-Whitney U test or Wilcoxon's signed-ranks test). The reason for also using non-parametric tests was the relatively small sample sizes, and that the data did not seem to satisfy in all cases the assumption of a normal distribution. The results for the parametric and the non-parametric tests differed only on very few occasions. We have therefore chosen only to present the results from the parametric tests. Pearson's correlation coefficient was used to determine the relationship between the experts' and the commuting participants' mean scores for the differences between ratings of inner urban and suburban environments.

The test-retest reproducibility was assessed using three types of analyses [38]. First, Student's paired t-test was used to assess the possibility of significant order effects, i.e. the significant changes in the mean between test and retest. Second, the standard error of measurement, i.e. the typical error for the difference between test and retest, was used, based on that the absolute sizes of the test-retest differences were of the same order of magnitude independent of the size of the ratings at test [39]. Third, the intraclass correlation coefficient (ICC) based on a one-way analysis of variance, along with 95% confidence intervals, was used. Ratings suggested by Landis and Koch [40] (< 0.00, 'poor'; 0.00-0.20, 'slight'; 0.21-0.40, 'fair'; 0.41-0.60, 'moderate'; 0.61-0.80, 'substantial', and 0.81-1.00 'almost perfect') were used as agreement levels when interpreting the ICC results. Furthermore, regression to the mean was analysed by linear regression. All items' scores, except *bicycle paths*, which has an 11-point scale, were used together.

Statistical analyses were performed using Statistical Package for the Social Sciences version 17.0 (SPSS Inc., Chicago, IL). A statistical level corresponding to at least p ≤ 0.05 has been used to indicate significance. The data from the 43-44 participants, in both inner urban and suburban environments, are used for all items twice. First, for the criterion-related validity: inner urban vs. suburban at test and retest. Second, for the reproducibility (order effect): test vs. retest in inner urban and suburban environments. The statistical implication of this is an increased chance of obtaining significant differences. Lowering the alpha level, and thereby compensating for the increased chance, would, however, be counterproductive in detecting possible order effects. We have therefore not done so. However, in relation to the comparison between inner urban and suburban environments, it is relevant to be restrictive in obtaining significant results since this is part of the validation strategy of the study. In that respect, we have therefore chosen a level of significance of p ≤ 0.025 (cf. Table 3, see commuting participants at test and retest).

#### Differences between men and women

The data were evaluated for gender differences among the commuting participants. Initially, we tested whether gender affected differences between ratings of inner urban and suburban environments (women, n = 16, and men, n = 28). This was the case in 2 out of 36 possibilities (18 items at test and retest, respectively). Thus, in general, there were no gender differences in this respect. The results pertaining to validity are therefore presented for men and women altogether.

Since previous studies [7,13,15] have shown gender differences, although few and small, in test-retest reproducibility concerning ratings of environments, we performed separate analyses in this area as well. First, we tested whether there were any significant gender differences in test and retest values, respectively (in total, women, n = 20, and men, n = 34). This was the case in 9 out of 72 possibilities (18 for inner urban and suburban environments and at test and retest, respectively). However, in none of the 72 cases were the male or the female mean values close to the response scale's minimal or maximal values. This allows for equal potentials to obtain test-retest differences of similar magnitude independently of gender. Thus, there were no risks for floor nor ceiling effects. Second, we tested whether gender affected differences between test and retest values. This was the case in 3 out of 36 possibilities (18 items in inner urban and suburban environments, respectively). Therefore, the results pertaining to test-retest reproducibility are also presented for men and women together.

## Results

### Criterion-related validity: differences between inner urban and suburban environments

The ratings of both the expert panel and commuting participants at test and retest show significantly higher values for the inner urban environments than for the suburban environments on the items: *exhaust fumes*, *noise *and *congestion: all types of vehicles*. The opposite was true for the item *greenery *(see Table 3). These findings correspond with the directions of the existing objective measures (see Methods).

**Table 3:** Ratings of environments by the expert panel and commuting participants at test and retest

	Expert panel (n = 22-24)			Commuting participants (n = 43-44)					
		
				Test			Retest		
					
Item	Inner urban mean ± SD	Suburban mean ± SD	t-test p-value	Inner urban mean ± SD	Suburban mean ± SD	t-test p-value	Inner urban mean ± SD	Suburban mean ± SD	t-test p-value
1. On the whole	8.21 ± 2.28	9.13 ± 2.33	0.219	9.43 ± 3.42	10.98 ± 2.80	0.001	8.86 ± 3.15	10.63 ± 2.68	0.000
2. Hinders or stimulates	7.79 ± 3.06	8.96 ± 2.49	0.071	9.98 ± 3.29	11.23 ± 2.57	0.004	9.18 ± 3.04	10.27 ± 2.86	0.004
3. Exhaust fumes	10.04 ± 2.60	7.50 ± 2.83	0.000	9.98 ± 2.80	7.91 ± 3.58	0.000	9.77 ± 3.09	7.32 ± 3.63	0.000
4. Noise	11.50 ± 2.18	9.45 ± 2.28	0.001	9.98 ± 2.77	8.50 ± 3.35	0.006	9.91 ± 2.44	8.52 ± 3.62	0.010
5. Flow of motor vehicles	12.09 ± 2.27	9.30 ± 3.02	0.000	12.27 ± 2.64	9.98 ± 3.73	0.000	11.41 ± 2.30	8.91 ± 3.84	0.000
6. Speeds of motor vehicles	9.00 ± 2.73	10.52 ± 2.41	0.006	8.95 ± 2.80	9.41 ± 2.68	0.098	9.25 ± 2.60	9.23 ± 2.81	0.964
7. Speeds of bicyclists	9.38 ± 2.99	10.62 ± 2.20	0.058	8.73 ± 2.73	9.11 ± 2.46	0.202	8.91 ± 2.68	8.98 ± 2.42	0.831
8. Congestion: all types of vehicles	11.92 ± 2.62	7.92 ± 2.45	0.000	10.61 ± 3.23	6.57 ± 2.92	0.000	10.30 ± 2.79	6.41 ± 3.14	0.000
9. Congestion: bicyclists	12.58 ± 2.06	7.33 ± 2.68	0.000	9.70 ± 3.59	5.41 ± 3.21	0.000	9.02 ± 3.69	5.75 ± 3.44	0.000
10. Conflicts	12.12 ± 1.92	8.58 ± 2.34	0.000	9.20 ± 3.98	4.98 ± 3.32	0.000	8.37 ± 3.77	5.74 ± 3.53	0.000
11. Bicycle paths*	6.42 ± 1.50	6.79 ± 1.18	0.372	6.93 ± 1.94	7.79 ± 2.35	0.058	6.84 ± 2.17	7.45 ± 2.50	0.222
12. Traffic: unsafe or safe	6.39 ± 2.46	9.43 ± 1.83	0.000	8.82 ± 3.42	12.00 ± 2.29	0.000	8.82 ± 3.37	11.41 ± 2.54	0.000
13. Greenery	5.58 ± 2.52	9.83 ± 2.18	0.000	7.48 ± 3.77	10.86 ± 2.81	0.000	7.57 ± 3.62	10.18 ± 2.81	0.000
14. Ugly or beautiful	10.58 ± 2.81	7.79 ± 2.70	0.002	11.20 ± 2.81	10.16 ± 3.41	0.073	10.73 ± 2.65	9.77 ± 3.06	0.061
15. Course of the route	10.50 ± 3.16	9.83 ± 3.04	0.409	7.50 ± 3.47	5.07 ± 3.55	0.000	7.11 ± 3.48	5.36 ± 2.98	0.002
16. Hilliness	7.29 ± 2.69	9.00 ± 1.96	0.005	4.77 ± 3.44	6.39 ± 3.92	0.012	5.39 ± 3.53	6.52 ± 3.62	0.015
17. Red lights	11.04 ± 3.37	8.54 ± 3.74	0.001	8.39 ± 3.86	4.48 ± 3.47	0.000	8.14 ± 3.83	5.16 ± 3.45	0.000
18. Short or long†	-	-	-	6.73 ± 2.17	6.86 ± 2.74	0.777	7.32 ± 1.90	7.11 ± 2.32	0.604

*Minimal value = 0, and maximal value = 10.

†Not assessed by the expert panel.

Mean scores on all items regarding ratings of inner urban and suburban environments for the expert panel and the commuting participants at test and retest are shown in Table 3. Significant differences were seen between ratings of inner urban and suburban environments in 12 of 17 items rated by the expert panel, and in 13 of 18 items rated by the commuting participants at both test and retest. A correspondence in both the significance and directions of the differences was noted in 10 of the 17 items for the two groups of raters.

**Figure 2:**
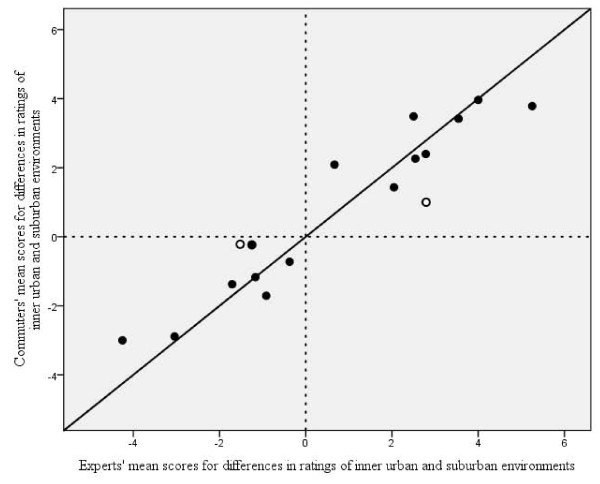
**The relationship of differences in perceptions of two environments rated by experts and active commuters**. The relationship between mean scores for the differences between perceptions of inner urban and suburban environments for the experts' and the commuters' test-retest means for 17 items. The diagonal line represents the line of identity. For both groups of raters, the mean values were either negative or positive and were therefore distributed in only two of the possible four fields of placement. The Pearson's correlation coefficient was 0.94. The symbol '○' denotes a significant difference in the size of the differences between the two groups of raters.

Mean scores for the differences between the inner urban and suburban environments for the expert panel and the commuting participants at test and retest, respectively, are shown in Table 4. There were only significant differences between the commuting participants at test and retest in 3 items. These scores were therefore combined to give a test-retest mean for each item, and compared with the ratings of the expert panel. The sizes and directions of the differences in ratings of inner urban and suburban environments corresponded well (*r *= 0.94) between the experts and the active commuters, and differed only significantly for 2 items (Figure 2).

**Table 4:** Differences between environments rated by the expert panel and commuting participants at test and retest

	Expert panel (n = 22-24)	Commuting participants (n = 43-44)				Difference commuters - experts	
			
Item	Mean ± SD	Test mean ± SD	Retest mean ± SD	t-test p-value	Test-retest mean ± SD*	Mean ± SEd**	t-test p-value
1. On the whole	-0.92 ± 3.55	-1.65 ± 2.86	-1.77 ± 2.74	0.771	-1.71 ± 2.48	-0.79 ± 0.82	0.339
2. Hinders or stimulates	-1.17 ± 3.02	-1.25 ± 2.76	-1.09 ± 2.36	0.671	-1.17 ± 2.25	0.00 ± 0.65	0.995
3. Exhaust fumes	2.54 ± 2.67	2.07 ± 3.02	2.45 ± 3.58	0.396	2.26 ± 2.96	-0.28 ± 0.73	0.701
4. Noise	2.04 ± 2.44	1.48 ± 3.39	1.39 ± 3.43	0.815	1.43 ± 3.16	-0.61 ± 0.77	0.428
5. Flow of motor vehicles	2.78 ± 2.81	2.30 ± 3.43	2.50 ± 3.95	0.623	2.40 ± 3.43	-0.38 ± 0.83	0.646
6. Speeds of motor vehicles	-1.52 ± 2.39	-0.45 ± 1.78	0.02 ± 3.34	0.371	-0.22 ± 2.03	1.31 ± 0.56	0.022
7. Speeds of bicyclists	-1.25 ± 3.07	-0.40 ± 2.00	-0.07 ± 2.13	0.212	-0.23 ± 1.89	1.02 ± 0.60	0.097
8. Congestion: all types of vehicles	4.00 ± 3.19	4.04 ± 3.63	3.89 ± 3.64	0.731	3.97 ± 3.30	-0.03 ± 0.82	0.967
9. Congestion: bicyclists	5.25 ± 3.07	4.30 ± 3.91	3.27 ± 3.64	0.017	3.78 ± 3.52	-1.47 ± 0.86	0.091
10. Conflicts	3.54 ± 2.47	4.21 ± 3.98	2.63 ± 2.95	0.006	3.42 ± 3.00	-0.12 ± 0.72	0.865
11. Bicycle paths†	-0.38 ± 2.02	-0.86 ± 2.89	-0.58 ± 3.32	0.493	-0.72 ± 2.82	-0.35 ± 0.65	0.598
12. Traffic: unsafe or safe	-3.04 ± 2.38	-3.18 ± 3.46	-2.59 ± 3.10	0.223	-2.89 ± 2.88	0.16 ± 0.70	0.823
13. Greenery	-4.25 ± 3.18	-3.39 ± 4.00	-2.61 ± 3.69	0.103	-3.00 ± 3.52	1.25 ± 0.86	0.153
14. Ugly or beautiful	2.79 ± 3.80	1.04 ± 3.78	0.95 ± 3.29	0.808	1.00 ± 3.32	-1.79 ± 0.89	0.047
15. Course of the route	0.67 ± 3.89	2.43 ± 3.77	1.75 ± 3.42	0.112	2.09 ± 3.32	1.42 ± 0.90	0.116
16. Hilliness	-1.71 ± 2.69	-1.61 ± 4.07	-1.14 ± 2.98	0.329	-1.38 ± 3.18	0.33 ± 0.77	0.665
17. Red lights	2.50 ± 3.28	4.00 ± 4.03	2.98 ± 3.53	0.041	3.49 ± 3.44	0.99 ± 0.86	0.256
18. Short or long‡	-	-0.14 ± 3.17	0.20 ± 2.59	0.387	-	-	-

*Test and retest scores combined to give a test-retest mean. For further explanation, see the text.

**SEd stands for standard error of difference.

†Minimal value = 0, and maximal value = 10.

‡Not assessed by the expert panel.

### Test-retest reproducibility of inner urban and suburban environments

The test-retest reproducibility results for each item regarding the inner urban environment are shown in Table 5. Order effects were seen in the items *hilliness*, *conflicts*, *congestion: bicyclists *and *hinders or stimulates*. The range of the typical errors was from 0.93 to 2.54. The range of the ICCs was from 'moderate' (0.42) to 'almost perfect' (0.87). Six items had a value of 0.41-0.60 ('moderate'), 10 items had a value of 0.61-0.80 ('substantial') and 2 items had a value of 0.81-1.00 ('almost perfect').

**Table 5:** Test-retest reproducibility of inner urban environment rated by the commuting participants (n = 52-53)

	Test		Retest		Test-retest difference			
			
Item	Mean ± SD	Min - max	Mean ± SD	Min - max	Mean ± SD	t-test p-value	Typical error	ICC (95% CI)*
1. On the whole	9.58 ± 3.25	3 - 15	9.06 ± 3.07	3 - 14	0.53 ± 2.14	0.078	1.51	0.76 (0.62 - 0.86)
2. Hinders or stimulates	10.02 ± 3.19	3 - 15	9.26 ± 2.97	2 - 15	0.75 ± 2.09	0.011	1.48	0.75 (0.60 - 0.84)
3. Exhaust fumes	9.92 ± 2.84	3 - 15	9.87 ± 2.96	3 - 15	0.06 ± 2.66	0.878	1.88	0.58 (0.38 - 0.74)
4. Noise	10.02 ± 2.74	4 - 15	9.96 ± 2.37	4 - 15	0.06 ± 2.78	0.883	1.96	0.42 (0.17 - 0.62)
5. Flow of motor vehicles	12.09 ± 2.68	4 - 15	11.51 ± 2.30	5 - 15	0.58 ± 2.26	0.066	1.60	0.57 (0.36 - 0.73)
6. Speeds of motor vehicles	8.92 ± 2.77	1 - 15	9.28 ± 2.54	4 - 14	-0.36 ± 2.63	0.326	1.86	0.51 (0.28 - 0.68)
7. Speeds of bicyclists	8.77 ± 2.65	4 - 15	8.94 ± 2.62	3 - 14	-0.17 ± 1.32	0.350	0.93	0.87 (0.79 - 0.93)
8. Congestion: all types of vehicles	10.60 ± 3.18	2 - 15	10.26 ± 2.80	3 - 15	0.34 ± 2.05	0.233	1.45	0.76 (0.63 - 0.86)
9. Congestion: bicyclists	9.72 ± 3.46	2 - 15	8.96 ± 3.51	1 - 14	0.75 ± 1.94	0.007	1.37	0.83 (0.72 - 0.90)
10. Conflicts	9.31 ± 3.97	1 - 15	8.50 ± 3.66	1 - 15	0.81 ± 2.77	0.041	1.96	0.72 (0.56 - 0.83)
11. Bicycle paths†	6.70 ± 2.07	2 - 10	6.72 ± 2.16	1 - 10	-0.02 ± 1.74	0.937	1.23	0.67 (0.49 - 0.79)
12. Traffic: unsafe or safe	8.89 ± 3.44	1 - 15	8.94 ± 3.30	3 - 15	-0.06 ± 2.75	0.881	1.94	0.67 (0.50 - 0.80)
13. Greenery	7.08 ± 3.82	1 - 15	7.45 ± 3.52	1 - 14	-0.38 ± 2.88	0.334	2.04	0.69 (0.52 - 0.81)
14. Ugly or beautiful	11.38 ± 2.68	5 - 15	11.04 ± 2.67	3 - 15	0.34 ± 2.17	0.261	1.53	0.67 (0.49 - 0.79)
15. Course of the route	7.34 ± 3.54	1 - 14	7.08 ± 3.50	1 - 14	0.26 ± 3.59	0.595	2.54	0.48 (0.25 - 0.67)
16. Hilliness	4.74 ± 3.29	1 - 12	5.62 ± 3.50	1 - 14	-0.89 ± 3.15	0.045	2.23	0.55 (0.33 - 0.71)
17. Red lights	8.29 ± 4.03	1 - 15	8.37 ± 3.76	1 - 15	-0.08 ± 3.48	0.874	2.46	0.61 (0.40 - 0.75)
18. Short or long	6.53 ± 2.22	2 - 10	6.94 ± 2.06	2 - 12	-0.42 ± 1.78	0.096	1.26	0.64 (0.46 - 0.78)

*Intraclass correlation coefficient with 95% confidence interval.

†Minimal value = 0, and maximal value = 10.

The test-retest reproducibility results for each item regarding the suburban environment are shown in Table 6. Order effects were seen in the items *flow of motor vehicles *and *hinders or stimulates*. The range of the typical errors was from 1.11 to 2.38. The range of the ICCs was from 'moderate' (0.46) to 'almost perfect' (0.82). Six items had a value of 0.41-0.60 ('moderate'), 11 items had a value of 0.61-0.80 ('substantial') and 1 item had a value in the range of 0.81-1.00 ('almost perfect').

**Table 6:** Test-retest reproducibility of suburban environment rated by the commuting participants (n = 44-45)

	Test		Retest		Test-retest difference			
			
Item	Mean ± SD	Min - max	Mean ± SD	Min - max	Mean ± SD	t-test p-value	Typical error	ICC (95% CI)*
1. On the whole	11.07 ± 2.86	4 - 15	10.73 ± 2.73	5 - 15	0.34 ± 2.23	0.316	1.58	0.68 (0.49 - 0.81)
2. Hinders or stimulates	11.31 ± 2.60	4 - 15	10.38 ± 2.91	3 - 15	0.93 ± 2.28	0.009	1.61	0.62 (0.40 - 0.77)
3. Exhaust fumes	7.78 ± 3.65	1 - 15	7.20 ± 3.67	1 - 15	0.58 ± 2.78	0.171	1.96	0.71 (0.52 - 0.83)
4. Noise	8.36 ± 3.45	1 - 14	8.38 ± 3.71	1 - 15	-0.02 ± 2.97	0.960	2.10	0.66 (0.46 - 0.80)
5. Flow of motor vehicles	9.80 ± 3.88	2 - 15	8.78 ± 3.90	2 - 15	1.02 ± 3.18	0.037	2.25	0.64 (0.44 - 0.79)
6. Speeds of motor vehicles	9.22 ± 2.93	1 - 15	9.07 ± 2.98	2 - 14	0.16 ± 3.10	0.738	2.19	0.46 (0.20 - 0.66)
7. Speeds of bicyclists	9.11 ± 2.46	5 - 15	8.95 ± 2.40	4 - 14	0.16 ± 1.57	0.505	1.11	0.79 (0.65 - 0.88)
8. Congestion: all types of vehicles	6.47 ± 2.97	1 - 13	6.31 ± 3.17	1 - 13	0.16 ± 2.99	0.729	2.11	0.53 (0.29 - 0.71)
9. Congestion: bicyclists	5.40 ± 3.17	1 - 12	5.67 ± 3.45	1 - 13	-0.27 ± 2.17	0.414	1.53	0.79 (0.65 - 0.88)
10. Conflicts	5.02 ± 3.28	1 - 13	5.75 ± 3.48	1 - 13	-0.73 ± 3.16	0.134	2.23	0.56 (0.31 - 0.73)
11. Bicycle paths†	7.82 ± 2.33	2 - 10	7.48 ± 2.51	2 - 10	0.34 ± 2.25	0.321	1.59	0.57 (0.33 - 0.74)
12. Traffic: unsafe or safe	12.04 ± 2.29	6 - 15	11.49 ± 2.56	6 - 15	0.56 ± 2.33	0.117	1.65	0.53 (0.28 - 0.71)
13. Greenery	10.93 ± 2.82	2 - 15	10.29 ± 2.87	2 - 15	0.64 ± 2.48	0.088	1.75	0.61 (0.38 - 0.76)
14. Ugly or beautiful	10.27 ± 3.45	4 - 15	9.89 ± 3.12	3 - 15	0.38 ± 1.97	0.205	1.39	0.82 (0.69 - 0.90)
15. Course of the route	4.98 ± 3.56	1 - 13	5.29 ± 2.99	1 - 11	-0.31 ± 2.69	0.441	1.90	0.67 (0.47 - 0.80)
16. Hilliness	6.27 ± 3.96	1 - 14	6.56 ± 3.59	1 - 13	-0.29 ± 3.37	0.568	2.38	0.61 (0.39 - 0.76)
17. Red lights	4.40 ± 3.47	1 - 15	4.98 ± 3.48	1 - 14	-0.58 ± 3.12	0.221	2.21	0.59 (0.37 - 0.75)
18. Short or long	6.89 ± 2.72	1 - 12	7.13 ± 2.30	1 - 10	-0.24 ± 2.06	0.430	1.46	0.67 (0.47 - 0.80)

*Intraclass correlation coefficient with 95% confidence interval.

†Minimal value = 0, and maximal value = 10.

Linear regression analyses of the test-retest differences (y-axis) in relation to the values at test (x-axis) showed expected regressions to the mean. The following equations were obtained for inner urban and suburban environments; y = -2.81 (-3.22 - -2.41) + 0.33 (0.28 - 0.37) x, and y = -2.19 (-2.59 - -1.80) + 0.28 (0.24 - 0.33) ×, (95% confidence interval), respectively.

## Discussion

This is, to our knowledge, the first report on the development of an environmental scale designed specifically to assess bicyclists' perceptions of their commuting route environments together with validity and reliability assessments. The overall results show considerable criterion-related validity and reasonable test-retest reproducibility.

What is the evidence for these conclusions? Since each active commuter has a specific route, the validity of their average perception of route environments is difficult to evaluate on an individual level. Instead, we have based the criterion-related validity assessment of the scale on whether or not some general differences between the inner urban and the suburban environments are reflected by differences in perceptions of those environments. This corresponds to the 'known group difference method' [33]. The first check for our test of the criterion-related validity was that existing objective differences between inner urban and suburban environments in Greater Stockholm, corresponding to our four items, *exhaust fumes*, *noise*, *congestion: all types of vehicles *and *greenery*, should be illuminated in differences in ratings of both the experts and the commuting participants. This was the case. In a way, this could be an excepted result. Nevertheless, taking into consideration the difficulty of validating perceptions of route environments, this represents a feasible and important first step. Note that, by no means, do the results signal non-discriminatory ratings of the items with objective differences; the average differences between the urban and suburban environments in *greenery *and *congestion: all types of vehicles *were, for experts and commuters, about 3 - 4 scale steps, but only 1.5 - 2.5 relating to *noise *and *exhaust fumes*. Thus, what might at first sight appear as rather evident and simple, may in the participants' ratings be captured in more intricate terms. Future studies using measurements that are more objective may further develop the understanding of validity issues related to the ACRES. The second check was that the differences in ratings of other items in relation to these environments should more or less show correspondence between the experts and the commuting participants. This was also observed. A correspondence between experts and commuters in both significance and directions of the differences was noted in 10 of the 17 items. There was also consistency between test and retest in the differences between inner urban and suburban environments among the commuters. This further strengthens the validity. The third check was that not only the directions, but also the sizes, of the differences in ratings of the environments by the participants should resemble, in general terms, those of the experts. Indeed, this was the case, as illustrated in Figure 2. In conclusion, the results of all our tests of criterion-related validity point in the same direction. Therefore, we regard the criterion-related validity as considerable.

In contrast to these checks of similarity, we had no expectations that the absolute levels of the ratings of the items would show high concordance between the experts and the commuters. This was so because the experts were asked to rate the overall environment of cyclists commuting as a whole, whereas the commuters were asked to rate their own self-chosen route environments. Furthermore, the ratings were done during different parts of the year and in different years.

There are many types of validity, and it can therefore be tested in different ways. Nevertheless, validity is rarely reported for questionnaires that have been developed to assess the neighbourhood environment associated with physical activity subjectively. Some validity results have been reported, however, for the NEWS [3,10,17-19]. Saelens et al. [10] assessed the construct validity of the NEWS using a design somewhat similar to ours. They used neighbourhoods characterized as having high or low so-called walkability for their validation. Their results showed differences between high- and low-walkability neighbourhoods measured with the NEWS. This finding is in line with our results showing that differences in environments can be assessed with self-reports.

Overall, the results indicate a reasonable test-retest reproducibility. A frequently used measure of reliability is the ICC. In the present study, the overall ICCs for both inner urban and suburban environments range from 0.42 to 0.87. This result is similar to findings from other reliability studies concerning questionnaires developed to assess neighbourhood environments believed to be associated with physical activity behaviours [3,7,9,10,12,13,15,16]. This similarity is interesting because the ACRES has 15-point response scales, compared to the other frequently used scales with fewer response alternatives. One expectation might be that more response alternatives would possibly result in lower reliability. Furthermore, the nature of the items assessed is somewhat changeable, e.g. the number of bicycle commuters in the bicycle paths may change considerably depending on weather conditions. Therefore, low test-retest values could reflect actual changes in the environments.

Interestingly, e.g. the item *congestion: bicyclists *shows an order effect but a high ICC value for the inner urban environment. Furthermore, the item *hinders or stimulates *shows an order effect, but substantial ICC values for both inner urban and suburban environments. This finding of contradictory indications of test-retest reproducibility is in line with Alexander et al. [7] who reported a high percentage agreement, but only fair ICC. It emphasizes the point of using several tests in the interest of understanding the nature of reproducibility and for comparability.

Several possible limitations of the present study need to be illuminated. First, considerations regarding the generalizability. Active commuters normally represent a small proportion of the population in larger cities. It is therefore difficult to use population-based random samples to study this group. In our case, the aim was to capture people who commuted in both inner urban and suburban environments, which made it even more difficult. Our solution, recruitment of participants at three different focal centres, passages between the suburban and the inner urban areas, as well as two other city centre recruitment places, most likely led to a sufficiently representative sample of active commuters during the part of the year studied. This population appears to be characterized by all-year-round active commuting. The frequency of commuting trips per week was approximately 8 and 5, respectively, during the assessment periods of November and December. During the summer half-year, there is an additional group of bicycle commuters. This group is, during this period, characterized by a high median frequency of commuting trips per week (predominantly about 8) [41]. The present findings of reproducibility therefore most probably also refer to this subpopulation of active commuters. It would, however, be useful to check the reproducibility and the criterion-related validity using different samples, e.g. active commuters with a lower yearly trip frequency [cf. [41]]. Second, the ratings of the commuting participants were collected mainly in November and December, 2005, and the ratings of the expert panel were collected in September and October, 2009. The compared ratings could therefore be based on somewhat different environments. However, only minor, if any, changes have occurred in Greater Stockholm leading to differences in route environments [cf. [32]]. Third, the possible lack of important items. One area, quality of the surface and surface maintenance for bicycling, was indicated by several of the experts as a factor that they felt was missing. This could, however, easily be added in a future version of the ACRES. Other items, such as crime safety and presence of pavements included in some questionnaires [cf. [11]], are not suitable for the circumstances in our study area, but they can also easily be added to the ACRES for use in other cultural contexts. Forth, there are numerous potential biases to consider when working with self-report questionnaires [42]. In line with people's capacity to discriminate on response scales, five to nine steps are ideal in most circumstances [cf. [42]]. The majority of our environmental items have, however, 15-point response scales. More steps than generally recommended were selected in an attempt to allow raters to make finer distinctions, and to facilitate discriminatory correlation studies. The scales were therefore strengthened by numbering the line with the entire range of values, i.e. whole numbers: 1 to 15, and by using neutral options in the middle, i.e. at number 8. The reasonable test-retest reproducibility and the distribution of responses, ranging from nearly minimum to maximum for all response scales (cf. Table 5 and 6), can be interpreted as support for the use of the 15-point scales.

The present study has several strengths. One is that the ACRES has been developed for the assessment of the individual route environment specifically and enables correlation studies between predictor variables and principally different outcome variables in relation to active commuting (e.g. traffic safety, hindrance or stimulation and levels of active commuting). Furthermore, most other questionnaires on physical activity and the environment define the measured environmental area as the local neighbourhood. However, the areas of individuals' environments for physical activity might extend further. Indeed, an important aim in the development of the ACRES was to create a scale with complete spatial matching between the environment and the physical activity variable. Another strength is that our participants were bicycle commuters. This is in line with the recommendation of Giles-Corti et al. [4] which emphasizes both the importance of studying specific physical activity behaviours and the specific environment within which the behaviour occurs. As regular bicycle commuters, our participants were probably very familiar with the route environments and therefore their perceptions might differ from those of non-commuters [22]. We believe, however, that the ACRES may well be used to study less regular commuters too. Furthermore, with a slightly modified version of the ACRES, non-commuters' perceptions could be studied. This may give a more comprehensive understanding of the route environment in relation to active commuting. Other important strengths are the validity tests, as well as the reliability tests of two different environments. Furthermore, some items emerged form the theory of space syntax, developed by researchers in the field of architecture and city planning in relation to active transport. To our knowledge, this has not been integrated in previous more extensive environmental scales that aim to study the relation between environment and physical activity. This adds to the construct and content validity of the ACRES.

## Conclusions

In conclusion, the ACRES demonstrates considerable criterion-related validity and reasonable test-retest reproducibility. Consequently, the results support the use of this environmental scale in future research to assess bicyclists' perceptions of different variables in their commuting route environments, and to further our knowledge of the potential relationship between these factors and active commuting behaviours.

## Competing interests

The authors declare that they have no competing interests.

## Authors' contributions

All authors contributed to the design of different parts of the study. ES and PS designed the scale and the reproducibility study, and ES was responsible for data acquisition. LW and PS designed the validity study, and LW was responsible for data acquisition. LW performed the statistical analyses and drafted the first version of the manuscript. PS drafted the manuscript, and supervised LW and ES as part of their PhD training. All authors read and approved the final manuscript.
